# The integrated social cognitive theory with the TAM model: The impact of M-learning in King Saud University art education

**DOI:** 10.3389/fpsyg.2022.1050532

**Published:** 2022-11-24

**Authors:** Abeer S. Almogren, Norah A. Aljammaz

**Affiliations:** Department of Art Education, College of Education, King Saud University, Riyadh, Saudi Arabia

**Keywords:** social cognitive theory, TAM model, students’ satisfaction, M-learning systems, higher education

## Abstract

Technology in higher education now includes a substantial amount of mobile learning (M-learning). M-learning also enables students to use the internet and technology for research, teamwork, and idea sharing. Additionally, in order to use M-learning systems, both students and teachers must accept M-learning. However, not enough research has been done in Saudi Arabia to determine how satisfied students are with their real use of mobile learning for educational purposes. As a result, the current study intends to investigate students’ behavioral intentions to utilize mobile learning, their happiness with the technology, as well as their impressions of how they actually use mobile learning systems. Therefore, this study aimed to develop a new model by integrating social cognition theory and the technology acceptance model to better understand the elements that influence the adoption of mobile learning in higher education (TAM). The majority of the information was gathered through a survey, with 412 university students’ randomly assigned questionnaires. The data analysis tools utilized were SPSS and Smart-PLS3.3.3. The studies proposed research model could, according to the study’s findings, account for 52.5% of the variation in how mobile learning systems were actually used. This information is crucial for understanding how social and educational technology factors affect the actual use of mobile learning systems. With only two hypotheses being rejected, this study created a new model that supported 16 of them. The findings indicated a beneficial relationship between 10 social and educational technology elements. The findings also indicated a favorable impact on students’ behavioral intentions to use and student happiness, which favorably impacts the actual use of M-learning in higher education. In order to improve students’ academic performance *via* mobile learning, social cognitive theory and the TAM model are combined as a consequence of the study’s empirical results. Therefore, we encourage students to collaborate with their colleagues at higher education institutions and use M-learning in their classrooms.

## Introduction

The market for mobile learning has shown rapid expansion in recent years. The usage of technological components has gained increased support from businesses and educational organizations. Students and teachers may now connect with upcoming learning possibilities, giving them a richer learning experience thanks in large part to technology in the mobile learning sector. Mobile learning is defined as possibilities for learning that are available anywhere and are conducted *via* mobile devices such cell phones, tablets, or tablet computers (Mutambara and Bayaga, 2021). Based on that, these tools enable students to access and engage with content on mobile devices anytime, anyplace (Pimmer et al., 2016; Al-Emran et al., 2018). Mobile learning often refers to learning while on the go (Behera and Purulia, 2013). This is further clarified by Kukulska-Hulme and Traxler (2005) who say that mobile learning enables people to carry out educational tasks without having to be in a particular place.

Utilizing transportable, light-weight mobile devices makes this possible. The impact of mobile devices on teaching, learning, and the relationship between formal and informal learning is also mentioned by the writers. In some ways, mobile learning is similar to online learning and distance learning, but it relies on learning across contexts using mobile devices, especially wireless mobile devices, which make it possible to learn anywhere, at any time (Georgieva et al., 2005).

Mobile learning, which offers the benefits of flexibility and mobility, is therefore seen as a novel idea in contemporary education and an extension of e-learning (Kumar Basak et al., 2018). M-learning has been found to benefit both students and teachers, improving both in-person and online student engagement (Badwelan et al., 2016).

M-learning has been the attention of many students and instructors, and various studies have examined how M-learning affects consumer usage (Mutambara and Bayaga, 2020; Saif et al., 2020). Even if smart apps are one of the main tools associated with learning, entertainment, and teaching (Hoi and Mu, 2021), there are now just a few suggestions on the sustainability and integration of mobile learning activities. Mobile devices and interaction, learning, and communication have a variety of linkages, according to Gikas and Grant (Almaiah et al., 2022). The use of collaboration tools for shared displays in M-learning has reportedly increased peer interactions in person (Almaiah et al., 2022). According to (Liaw et al., 2010) emphasized the benefits of adopting them, such as a rise in student satisfaction (SS), the encouragement of learner autonomy, the growth of student engagement, and improvements to system efficiency.

M-learning technologies have been employed in collaborative learning environments, according to (Dai et al., 2012). The findings indicate that problematic learning pedagogies have had a mostly positive effect, leading teachers to have more faith in and adoption of alternative teaching methods. The implementation of M-learning reveals social disparities among users (Alghazi et al., 2020). According to Shin and Kang (2015), m-learning has the ability to enhance interactions and collaboration between teachers and learners. Mobile technologies and social media play a crucial role in enabling and supporting interactive information sharing, interchange, user-centered design, and collaboration through social applications, file transfer, tagging, social media, blogs, wikis, and RSS (Sayaf et al., 2021).

However, other people may use social media for tasks including engaging with students through official Facebook, Skype, and blogs as well as scheduling exams, quizzes, and SMS messaging (Ada et al., 2017). On the other hand, some students may want to think about using mobile applications for studying, calendaring, uploading educational materials, engaging in peer discussion, sharing files, and taking tests and quizzes (Asghar et al., 2021). We used these platforms’ social media and mobile technology which learners are indeed accustomed to using on a regular basis to effectively engage students with feedback (Al-Rahmi et al., 2018a).

By implementing carefully thought-out mobile learning activities that might persuade students to participate in them, this is done in the hopes that students would transition from being passive to effective learning (Wang et al., 2009). Due to the suddenness and novelty of the problem, however, incorporating mobile learning using social media will have more adverse than favorable effects on students (Lim, 2020). For instance, because to the pervasiveness and social nature of mobile social media, students can check their friends’ posts and contact with one another whenever they want on the same platform that they are studying on. Technical and non-technical obstacles must yet be removed, especially in order for students to use and adopt M-learning (Almaiah et al., 2019).

Research has shown that m-learning is still a concern (Al-Emran et al., 2018; Kumar Basak et al., 2018). Additionally, the demands and expectations of M-learning clients are not well understood by current academics and mobile carriers. In fact, a crucial step in ensuring the system’s successful deployment in higher education is student acceptance of M-learning (Aremu and Adeoluwa, 2021).

Therefore, comprehension and identification are crucial factors in determining whether students accept M-learning systems. Additionally, the time and effort required for the deployment of any information system are expensive. To assure a system’s sustainability viability, information system researchers are constantly attempting to determine what factors influence a system’s adoption (Alamri et al., 2020).

Both teachers and students preferred M-learning for education, according to Sophonhiranrak (2021). Higher education institutions have made significant investments in m-learning initiatives, yet the majority of these initiatives continue to fall short of the anticipated system advantages (Althunibat et al., 2021). Dedicated research have demonstrated that for m-learning technology to be successful, pupils must fully accept it; else, the outcome would be failure (Sophea et al., 2021). In a different study, it was found that students’ adoption of m-learning technology affects its effectiveness in the learning environment (Almaiah et al., 2019). This kind of research is important for designers and developers of m-learning systems because it can help students make the most of this type of learning technology (Almaiah et al., 2019).

Additionally, m-learning programs provide university students a number of advantages, albeit usage and acceptance of m-learning systems vary from institution to institution (Almaiah et al., 2019).

The results of the literature review indicate that university students’ acceptance rates have remained low (Almaiah et al., 2019). Other research (Criollo-C et al., 2021) ignored quality issues as playing a key influence in the success of m-learning systems and their appraisal, whilst some other studies (Alshurideh et al., 2019) blamed the low level of m-learning system use and adoption among students on the low quality of m-learning systems and services. The failure of such systems to meet the needs and demands of students was addressed in other research as well. Although the Saudi Arabian Ministry of Education mandated the adoption of new technology in academic institutions, mobile learning in education is still a relatively undeveloped technology, and there is little research on the subject (Al Harthi, 2018).

The adoption of mobile learning in formal education was supported by the governments of the countries in the Arabian Gulf. The attitudes of students and teachers regarding this type of education, however, are poorly understood. Therefore, research on the perspectives and acceptance of mobile learning as a new pedagogical strategy at Arab universities is necessary (Al-Emran et al., 2018).

So, according to this study, all students in higher education institutions should be able to use new technology, including mobile devices, as instruments for gathering, researching, managing, accessing, organizing, and evaluating information. However, the majority of the research in this field has focused on mobile learning for informal self-education. In Saudi Arabia, mobile learning is typically related to students learning outside of the classroom (Almaiah et al., 2022).

Furthermore, there aren’t many research on “technology adoption” in higher education in Saudi Arabia, particularly when it comes to lecturers’ perceptions of mobile learning (Al-Hamad et al., 2021). This is explained by the idea that pupils could utilize them for things other than studying and become sidetracked from “real” learning as a result. Some Saudi colleges have decided to forbid the use of mobile devices as a result of this concern (Alsidrah, 2022).

Even though the majority of teachers are fairly wary about employing mobile devices in this setting, we still think there is a need to integrate them efficiently into Saudi classrooms. Therefore, by identifying these gaps, university students can better comprehend how M-learning affects their academic achievement. However, no prior study has looked at how satisfied students are with M-learning and how eager they are to use it for digital learning in Saudi Arabia’s higher education sector.

This study included social cognitive theory and TAM to examine students’ satisfaction with and actual use of M-learning systems in higher education. In order to understand the actual M-learning usage among Saudi Arabian university students, this study also attempted to develop a novel model and conduct a confirmatory factor analysis.

Thus, the contribution of this study is to develop a new model and analyze students’ behavioral intentions and actual use of mobile learning for educational purposes. As a result, the current study intends to investigate students’ behavioral intentions to utilize mobile learning, their happiness with the technology, as well as their impressions of how they actually use mobile learning systems. Therefore, this study aimed to develop a new model by integrating social cognition theory and the technology acceptance model to better understand the elements that influence the adoption of mobile learning in higher education (TAM).

## Mobile use and acceptance in Saudi education

Universities in Saudi Arabia have shown a marked rise in interest in mobile learning over the past 10 years (Abdulrahman and Benkhelifa, 2017). Many things, including the accessibility of wireless networks and the explosive development of mobile technology, are blamed for this tendency (Hoi and Mu, 2021). Additionally, due to affordability, the Saudi public has embraced the Internet and smartphones, with 28.8 million users in 2019 (Statista, 2020). In order to use submultiple mobile applications for teaching and learning, academic personnel and educators must do so. Many Saudi Arabian universities, like King Abdul-Aziz University in Jeddah, the Saudi Electronic University, and Albaha University, have made crucial investments in mobile learning to date (Alkhaldi and Abualkishik, 2019). The Saudi government has created the necessary infrastructure as a result for initiatives like the Saudi Digital Library (Taala et al., 2019) and the National Centre for E-learning and Distance Learning (Gupta et al., 2021). Due to the global COVID-19 dilemma, Saudi Arabia is currently seeing an increase in the utilization of educational technology like M-learning and m-learning (Alarifi, 2020). Therefore, Saudi institutions have shifted their operations to the platforms made available by various educational technologies so that students can receive educational material while they self-isolate at home. These include learning management systems (LMSs), which may be utilized on a variety of electronic devices, such as computers, tablets, and/or smartphones, and which can be accessed and browsed. When Alturki and Aldraiweesh (2022) looked into how students felt about utilizing mobile learning and how they behaved, they found that it had a positive impact on how mobile learning was really used in Saudi Arabia’s higher education system during the COVID-19 pandemic. AlEid (2019) used a semi-experimental methodology to explore the usage of mobile devices in learning at Princess Nourah University in Saudi Arabia. Overall, the research results confirmed that the adoption of mobile learning had a significant impact on learners’ perceptions. The study also showed that the availability of the Internet, human resources, and the readiness of teachers and pupils to use it are all necessary for the success of mobile learning. Additionally, Al-Fahad (2009) performed a study on female undergraduate students at King Saud University to find out how they felt about the effectiveness of mobile learning. The results imply that having access to mobile learning would increase student retention and enhance their educational experience. Saleem (2017) conducted research on the application of mobile learning for the teaching of English at Taibah University in Saudi Arabia more recently, and the findings showed that m-learning might improve self-learning and offer practice chances. Therefore, these studies came to the conclusion that m-learning can enhance the teaching and learning process. M-learning aids in facilitating and promoting student acceptance of in Saudi universities (Almaiah et al., 2019).

## Research hypotheses and theoretical model

**Figure 1:**
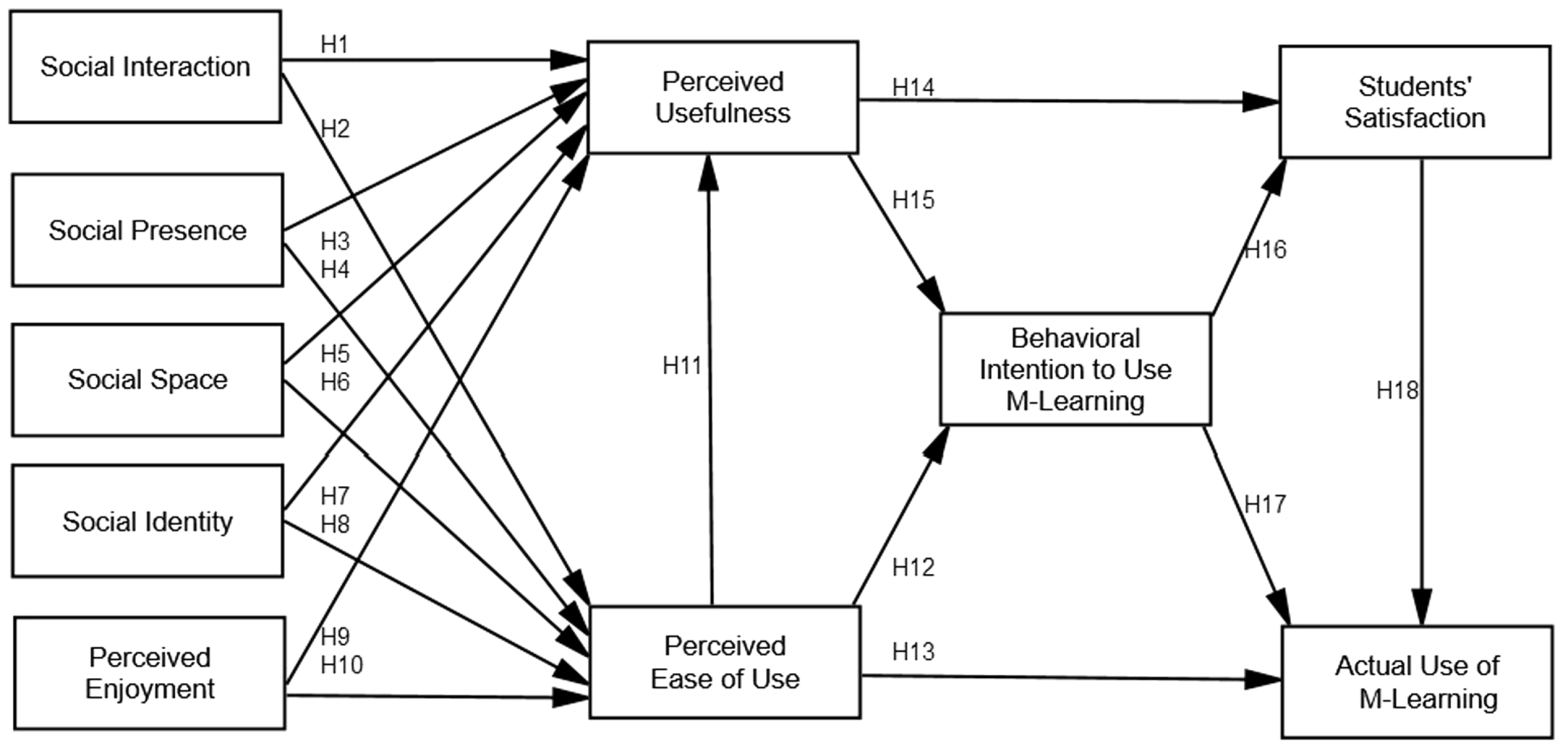
Research model.

According to social cognitive theory, people are active participants in their lives rather than passive recipients of environmental events-driven changes in their brains. People employ their sensory, motor, and mental systems as tools to complete the activities and achieve the objectives that give their life direction and significance (Harre and Gillet, 1994). The emergent interactive agency concept is supported by social cognitive theory (Bandura, 1997). People are neither mechanical carriers of animating environmental stimuli nor autonomous actors. In contrast to immaterial substances existing outside of neural systems, mental events are actually brain activity. Materialism does not, however, necessitate reductionism. Thought processes are emergent brain activity in a non-dualistic mentalism that are not ontologically reducible (Sperry, 1993). Social support and perceived social efficacy both influence human adaptability and transformation in both directions. Social support does not appear on its own, waiting to protect overworked individuals from pressures. Instead, individuals must seek out and build strong relationships for themselves that they can keep. When compared to people who doubt their social skills, those with high perceived social efficacy create circumstances that are more supportive of themselves (Holahan and Holahan, 1987a). A particularly promising method for gauging user attitudes and willingness to employ computer technology is the technology adoption model (TAM) (Davis et al., 1989; Vankatesh and Davis, 1996). According to several researches (Moafa et al., 2018), learner attitudes toward a particular technology are determined by user expectations of simplicity, usability, enjoyment, attitude toward usage, behavior that affects satisfaction with use, and actual use of the M-learning system. Based on earlier research on the TAM model (Davis et al., 1989; Al-Rahmi W.M. et al., 2021), this study offers 18 hypotheses about how M-learning may impact SS and actual use of M-learning in higher education. Therefore, to evaluate the students’ happiness and real use of the M-learning system in higher education, the integrated social cognitive theory and TAM to the adoption of technology are applied, as shown in Figure 1.

### Social interaction

When instructors employ tactics to promote interpersonal encouragement and social inclusion, this interaction between students and instructors is referred to as “social interaction” (Jung et al., 2002). Three forms of student, student, and ensuring that the educational are classified by Lonn et al. (2011). Learner-to-learner exchanges take place in a virtual environment whether or not teachers are present (Almaiah et al., 2022). When students gain access to information *via* a number of channels, such as social media and online courses, their perceptions of their academic accomplishment and involvement will rise (Ansari and Khan, 2020). The term “learner-instructor interaction” refers to the exchange of information, provision of appropriate assistance, clarification of student misunderstandings, and escalation of student excitement (Lonn et al., 2011). These three distinct social contact kinds are essential for assessing SS. Learning becomes more fun when various sorts of collaboration are implemented in the setting (Miyazoe and Anderson, 2010). There might be many points of contact by include extracurricular activities in the academic program. Even while student–student connection is required for online SS, the frequency, quality, and promptness of student-instructor interaction are the most crucial factors in predicting SS (Penney, 2020). In a study of 120 exceptional education nursing students, Thurmond et al. (2002) discovered that having a well-known instructor, receiving an instructor response fast, and choosing the evaluation technique were all associated to SS. These findings highlight the role of enjoyment in online learning as well as the importance of student-instructor interaction in enhancing student performance. Additionally, during the COVID-19 epidemic, it was discovered that perceived interaction and self-efficacy played the biggest roles in determining perceived utility and ease of use, which in turn influenced students’ intentions and happiness with e-learning (Gurban and Almogren, 2022). Additionally, a study by Almogren (2022) found that perceptions of usefulness and usability during the COVID-19 Pandemic had a statistically significant impact on behavior intent, actual blackboard usage, and Online learning interaction in art education classes. Additionally, Online connection quality traits influence how social media users interact and interaction learning (Sadiq et al., 2022). As a result, the following hypotheses are proposed in this research:

*H1*: Social interaction will have a positive effect on perceived usefulness.

*H2*: Social interaction will have a positive effect on perceived ease of use.

### Social presence

The definition and application of the phrase “social presence” are still up for discussion. By interacting with and receiving support from academics and office staff, students in our study were able to develop a sense of social presence, which can be characterized as how they perceive. In order to increase social presence and student retention in online teaching and learning, engagement tactics may also be implemented (Joksimović et al., 2015). These include of chances for self-evaluation, quick responses, accessibility, and greater chances for social contact in the classroom. Early studies on social presence stressed the value of students’ emotional relationships. In virtual learning settings, social presence is a crucial indicator of satisfaction and perceived learning (Swan and Shih, 2005). Baber, facial expressions, gestures, verbal tonality and pace, salutations, allusions to groups, acceptance, and direction increase student pleasure and perceived learning. The manner in which teachers interact with online educational classes, including their speech patterns, facial expressions, and the success of their engagements with them, all influence how students feel a sense of social presence (Akour et al., 2021). Teachers and students need to interact in order to promote critical thinking and ensure inclusivity in order to bring social presence to learning (Zhang et al., 2020). Kehrwald (2008) contends that interactions do not adequately describe pupils’ social presence. However, relational presence and the capacity to forge bonds and maintain continuing meaningful involvement can be used to gauge social presence. As a result, the following hypotheses are proposed in this research:

*H3*: Social presence will have a positive effect on perceived usefulness.

*H4*: Social presence will have a positive effect on perceived ease of use.

### Social space

Social ties between group members make up the social space. It consists of collections of values and standards, laws and obligations, convictions, and aspirations (Kreijns et al., 2004). Social space influences social interaction because of the members’ mutual trust and sense of belonging, which opens up opportunities for critical dialogue where open speech is neither offensive nor destructive. Information is freely exchanged, which improves adherence to the group’s goals and raises general satisfaction. In conclusion, “a healthy social space inside the group contributes to a pleasant social climate/online atmosphere,” according to Kreijns et al. (2004). Social space, sociability, and social presence are three interrelated ideas that cannot exist separately. When they collaborate, they have an impact on how social engagement in groups is established and maintained. Low sociability in a group negatively affects the creation of social space, according to research by Kreijns et al. (2004). Sociability adds to social space even though the two categories include various characteristics of interpersonal interactions in groups (Sjølie et al., 2022). It is believed that the community participants have an impact on how social space develops during asynchronous online talks (Shea et al., 2022). Members might opt to use the advantages of the learning environment or adhere to the group’s goals (i.e., sociability). Uncertainty exists regarding the aspects of social places that affect how people perceive them. Most social presence scholars utilize social presence theory, which merges the three components into a single “social presence” idea, because they are not familiar with the concepts of social space and sociability. As a result, the following hypotheses are proposed in this research:

*H5*: Social space will have a positive effect on perceived usefulness.

*H6*: Social space will have a positive effect on perceived ease of use.

### Social identity

The social identity includes both the self-categorization theory and the social identity theory (Turner et al., 1987). A person’s social identity may be referred to as their self-concept taking into consideration their membership in a social group (Turner et al., 1987). There are people who self-identify as belonging to a variety of social classes or groupings (Lund Dean and Jolly, 2012). To organize and situate themselves in their social settings, individuals employ categories (Kim et al., 2010), a social and relative approach that results in the identification of in-groups and out-groups (Jungert, 2013). The efficiency of online learning has an impact on social identities, according to a study by (Mingfang and Qi, 2018) that empirically examined the connection between students’ social identities and that interaction. Their research also highlights the need to strengthen students’ social identities in order to increase online learning outcomes and satisfaction. Social ties within a group, as well as individual students’ dedication to learning, academic achievement, and contentment with their curriculum and structure, all improve as a result of social identification, which boosts in-group homogeneity (Ashforth and Saks, 1996). Students who meet their educational purposes are more likely to be pleased with their course work and school (Wilkins and Epps, 2011). Since education is an identity experience that shapes a person’s capabilities, learning and social identification are strongly intertwined (Wenger, 1999). When they initially start in college, students have an academic self-concept, or a belief in their own academic abilities. The academic self-construct of students with high high school grade point averages is associated with exceptional goal attainment (Alenezi, 2022). Students who enroll in postgraduate programs and have a history of successful employment, such as as junior or midrange managers, share common social identity characteristics (Wortham, 2004). One’s perception of oneself as a “proven” manager may have a key role in their personality and have an impact on how they interact with students and teachers. As a result, the following hypotheses are proposed in this research:

*H7*: Social identity will have a positive effect on perceived usefulness.

*H8*: Social identity will have a positive effect on perceived ease of use.

### Perceived enjoyment

Regardless of any potential negative outcomes, perceived enjoyment refers to how fun students perceive certain activities or services to be (Van der Heijden, 2004). Therefore, in the current analysis, perceived satisfaction is defined as the enjoyment felt by learners as a result of using the M-learning approach in a way that enhances their learning experiences. According to (Eshnazarova and Katayeva, 2021), perceived pleasure might indicate a person’s behavioral intention to use information technologies. When it comes to learning, a student’s subjective sensations of fulfillment, relaxation, enjoyment, and a positive overall experience frequently play key roles in explaining the acceptance and usage behavior of e-user learning (Lutfi et al., 2022). The (Van der Heijden, 2004) study, which suggested that intrinsic motivators like perceived enjoyment could affect a user’s use of information systems like M-learning, provided evidence in support of this. The findings demonstrated that perceived enjoyment had a substantial influence on the student’s intention to use mobile learning. As a result, the following hypotheses are proposed in this research:

*H9*: Perceived enjoyment will have a positive effect on perceived usefulness.

*H10*: Perceived enjoyment will have a positive effect on perceived ease of use.

### Perceived ease of use

Perceived ease of use, one of the key elements of the original TAM, is characterized as the extent to which learners perceive using M-learning would be straightforward. Perceived ease of use (Davis et al., 1989) is the degree to which a person expects finding a specific system to be straightforward to use, and it is crucial for the future acceptability of revolutionary tech applications (Venkatesh, 2000). The choice to employ M-learning has been shown to be influenced by perceived ease of use in some earlier research (Al-Rahmi A.M. et al., 2021). As a result, perceived simplicity of use improves the possibility that the M-learning system will be employed, which in turn enhances that likelihood. Indirect influences on the propensity to utilize M-learning are also believed to come from perceived utility and perceived ease of use (Al-Rahmi A.M. et al., 2021). Additionally, it is anticipated that user intentions would be indirectly influenced by the perceived ease of use and utility of M-learning. According to (Hoi and Mu, 2021), PEU is simple for a client to employ in the context of M-learning. The workload for instructors increases when they use M-learning, even if they do not use the M-learning technology (Hoi and Mu, 2021). A difficult-to-use management system may have an effect on attitudes, utility assessments, and behavioral intentions in the early phases of system adoption, according to a claim made by Davis et al. (1989). As a result, the following hypotheses are proposed in this research:

*H11*: Perceived ease of use will have a positive effect on perceived usefulness.

*H12*: Perceived ease of use will have a positive effect on behavioral intention to use M-learning.

*H13*: Perceived ease of use will have a positive effect on actual use of M-learning.

### Perceived usefulness

The student level’s perception of the usefulness was described as their expectation that using M-learning will improve performance. Users of IS in the 21st century are able to adopt more innovative and user-friendly developments that allow them more independence thanks to perceived utility, which is a significant predictor of purpose (Al-Rahmi et al., 2017). It was discovered that the decision to employ M-learning services was significantly influenced by perceived usefulness (Kumar Basak et al., 2018). As a result, the likelihood of using the M-learning system increases with the perceived value of the system and the optimism with which it is intended to be used. The M-learning PU encourages positive behavior intentions and enhances M-learning utilization on the parts of students and trainers (Al-Rahmi et al., 2019). According to reports, the PU, which is also a very good predictor of both SS and BI (Al-Rahmi et al., 2019) according to current M-learning research (Sánchez-Prieto et al., 2019), had an impact on the original information system TAM, as well as the depending on the selection and purpose. As a result, the following hypotheses are proposed in this research:

*H14*: Perceived usefulness will have a positive effect on students’ satisfaction.

*H15*: Perceived usefulness will have a positive effect on behavioral intention to use M-learning.

### Behavior intention to use M-learning

The likelihood that a person will utilize an information system and educational technology is the key dependent variable identified in research conducted since the TAM and is known as the intention to use behavior. According to Alowayr and Al-Azawei (2021) and Ullah et al. (2021), it would be more crucial to employ technology when the amount of such activity linked with its use was higher. The TAM was used to include behavioral intention, which is defined as students’ intentions to use M-learning. In this study, it is anticipated that the behavioral goal and actual M-learning use will be statistically related. According to earlier research, students’ attitudes toward using M-learning were substantially correlated with their pleasure and actual usage of technology, particularly M-learning (Teo et al., 2019). Aim is important while using current technologies in practice (Davis et al., 1989). Several scholars have looked at the connection between M-intended learning’s application and actual use in the acceptance area (Al-Rahmi et al., 2015a). Venkatesh et al. (2003) provides evidence supporting the causal relationship between use intention and usage. In light of this research, it was concluded that the intended usage had a positive impact on how M-learning was actually used. The most important piece of acceptance technology for students to evaluate the acceptability of M-learning is likewise acknowledged to be the BI (Al-Rahmi et al., 2015b). The BI has a favorable effect on the usage of M-learning, according to studies on the subject (Buabeng-Andoh, 2018). As a result, the following hypotheses are proposed in this research:

*H16*: Behavioral intention to use will have a positive effect on students’ satisfaction.

*H17*: Behavioral intention to use will have a positive effect on actual use of M-learning.

### Students’ satisfaction

In terms of their overall perception of educational technology, individuals’ expectations of satisfaction are defined as the extent to which their requirements, priorities, and wishes have been adequately realized (Sánchez-Franco, 2009; Wang et al., 2009). Several studies have shown that satisfaction significantly increases one’s likelihood of using M-learning services (Ansari and Khan, 2020). Satisfaction has been shown to have a significant favorable effect on actual use as well. According to Al-Rahmi et al. (2015c) study’s, contentment has a positive impact on how the M-learning system is really used. It was therefore thought that in the context of this trial, pleasure had a favorable effect on both the desire to utilize and the actual utilization of M-learning. Students usually discovered that users of e-learning services are content to use them as intended (Liu et al., 2021). Increased user intention to employ M-learning is aided by improved user satisfaction (Shin and Kang, 2015). Additionally, it was discovered that satisfaction significantly influenced how successfully M-learning was used (Liaw et al., 2010). According to Liu et al. (2021), contentment had a positive impact on actual e-learning system usage. This study therefore predicted that BI and the actual application of M-learning would be advantageous. As a result, the following hypotheses are proposed in this research:

*H18*: Students’ satisfaction will have a positive effect on actual use of M-learning.

### Actual use of M-learning

The higher education system is currently going through a constant process of change, and colleges must adapt to the needs, expectations, and demands of their students. University operations are also heavily influenced by digital technology and M-learning platforms, with these institutions investing more and more in online systems and tools (Al-Rahmi et al., 2018b). The development of innovative M-learning platforms, however, to enhance and facilitate both teaching and learning is one of universities’ major problems in the technological era (Nikolopoulou et al., 2021). M-learning offers a variety of opportunities for exchanging information and uploading documents in different formats, which contributes to and nurtures the learning-teaching process in many ways. Because it is a web-based framework, no additional resources need to be deployed, and once the material is published, users can access it whenever they want (Shodipe and Ohanu, 2021). Due to the unique scenario that the pandemic has caused, experts are now very interested in the impact of the epidemic on education, universities, teachers, and students. When Allo looked into what students thought about online learning, she discovered that they had a favorable view toward it and felt it to be advantageous and practical during the pandemic-induced crisis (Allo, 2020). In contrast to the self-reported usage of students’ technology, the latter focuses on the actual use of mobile M-learning devices in schools, which can be impacted by response distortions (Heflin et al., 2017). The technique of education (learning) through social media using an user’s personal mobile devices, such as tablets and smartphones to access learning materials through mobile apps, human activities, and online educational resources is known as mobile learning, also referred to as “M-learning” or “M-learning.” It is flexible and gives students and learners access to education at any time and from any location (Sharples et al., 2009; Kukulska-Hulme, 2010). Finding out how satisfied college students are with their behavioral intention to utilize mobile learning as well as what they think about how they really use it are the goals of the current study.

## Research methodology

### Study design

This research conducted a survey of students at King Saud University to see how they use M-learning for both teaching and learning. To successfully achieve the study’s goals, the analysis was divided into two sections. First, information was acquired from university students utilizing a questionnaire (see Appendix). The study examined opinions about and actual use of M-learning, as well as how it impacts higher education. Students in higher education who participated in this survey comprised both undergraduates and university graduates. The responders were from a range of art education school, as well as the fields of engineering and social science. Some of the research participants are now using the M-learning system for learning, so we might be able to gain their help with the survey questions. The survey used a Likert scale with a maximum of five points. The five-point Likert scale is thought to be less accurate than this one (Joo et al., 2014). We completed the next phase of our investigation. The data was analysed using SPSS-24 and Smart-PLS 3.3 for Structural Equation Modeling. Concept validity, convergent validity, and discriminant validity of the structural model proposed for this form (Hair et al., 2019) were investigated. The proposed model, which has five components social interaction, social presence, social space, social identity, and emotional happiness as hanging variables is depicted in Figure 1.Perceived benefit, perceived ease of use, and behavior intention to employ M-learning as a mediator variable Additionally, there were two dependent variables: real M-learning utilization and SS. For the 10 constructs that will be utilized to determine how successfully students are utilizing M-learning in Saudi Arabia’s higher education system, this study generates 18 hypotheses.

### The measurement of variables and analysis software used

Ethical review and approval were waived for this study due to the adoption of a questionnaire from previous research. We also distributed the questionnaire to the students we teach, as well as other classes at the same university. Therefore, all the students who answered the questionnaire agreed once they responded. And those who did not agree to respond to the questionnaire were excluded. As shown in Table 1, a survey instrument was used to accomplish the research goals through a thorough analysis. There were 10 constructions and 38 indicators total. First, dependent variables, specifically social contact, were suggested with the creation of three items as advised by Wei and Chen (2012). In order to develop social presence, four items were suggested by Cobb (2009). Additionally, the establishment of four things in the social space was suggested by Kreijns et al. (2004). The establishment of three items as suggested by Zeng et al. (2009); Abdullah et al. (2016) was also used to propose social identity and the establishment of three items as suggested by Zeng et al. (2009); Abdullah et al. (2016), respectively, for perceived enjoyment. Perceived usefulness, perceived usability, and behavior intention to apply M-learning were proposed as the four items for each of the mediator factors by Ratna and Mehra (2015). Additionally, dependent variables, specifically student contentment, were proposed with the formation of five items as indicated by Cobb (2009), and actual M-learning utilization was supplied with the establishment of four items as advised by Ratna and Mehra (2015). Data analysis methods included partial least squares structural equation modeling (PLS-SEM). Utilizing the Smart-PLS 3.3.3 application, measurement and structural models were assessed in this study. The accuracy and dependability of the data were assessed as they were being used to compute the measurement model. In this research reported convergent and discriminant validity to evaluate the data’s validity. Cross-loading and the Fornell-Larcker criterion were utilized to address the discriminant validity, and an average variance extracted (AVE) formula with a value of 0.500 was used to define the convergent validity. To rate the dependability of the data, an internal consistency reliability approach was used. Composite Reliability (CR) and Cronbach’s Alpha (CA); both values should be more than 0.700; are dependability measurements. This research used the path coefficient, *t*-value, and value of *p* to report the relationship’s significance for the assessment model.

**Table 1:** Data collection and demographic analysis.

Gender	Frequency	Percent
Male	203	49.3
Female	209	50.7
Total	412	100.0
**Age**	**Frequency**	**Percent**
18–21	263	63.8
22–25	87	21.1
26–29	21	5.1
30–33	16	3.9
>34	25	6.1
Total	412	100.0
**Level of education**	**Frequency**	**Percent**
Undergraduate	342	83.0
Postgraduate	70	17.0
Total	412	100.0
**Specialization**	**Frequency**	**Percent**
Science and technology	83	20.1
Engineering	44	10.7
Art education	285	69.2
Total	412	100.0

### Data collection and demographic analysis

A total of 521 questionnaires were manually distributed and only 438, or (84.06%), were returned to the researchers. After excluding 9 incomplete questionnaires, and 7 of which were of missing data, 10 were outliners. Thus, the total number of valid questionnaires was 412 after this exclusion. 412 questionnaires were given out to students at King Saud University in order to conduct the study. A conceptual model for the study was created utilizing the social cognitive theory and the TAM model in order to monitor the students’ satisfaction and practical use of M-learning for educational purposes. In order to ascertain the behavior intention to use M-learning, as well as to ascertain the students’ happiness and actual usage of M-learning in a higher education environment, this study analyzed the students’ perspectives on the use of M-learning. University students were given a questionnaire and asked to reply anonymously about how mobile learning is used in education and how that has changed how M-learning is used in sustainable learning strategies. Structural equation modeling was utilized to evaluate the data along with IBM SPSS Statistics version 26 and Smart-PLS 3.3.3.There was a total of 412 surveys returned, 203 (49.3%) was from male students, and 209 (50.7%) was from female students. Next factor regarding to the age of the students, 263 (63.8%) was range between 18 and 21 years old because the majority of the respond from undergraduate level, 87 (21.1%) was range between 22 and 25 years old, 21 (5.1%) was range between 26 and 29 years old, 16 (3.9%) was range between 30 and 33 years old, and 25 (6.1%) was more than 34 years old. The level of education 342 (83.0%) was from undergraduate students, and 70 (17.0%) was from postgraduate students. The specialization of study 285 (69.2%) was collected from art education, 83 (20.1%) was collected from science and technology, and 44 (10.7%) was collected from engineering (see Table 1).

## Results and analysis

Least squares in part SEM (PLS-SEM) (Hair et al., 2019) has recently had a good effect on research output, and it is still being employed more and more in many domains, including marketing studies (Kwiatek et al., 2020), recommender systems research (Mican et al., 2020), and acceptance of health systems (Ho et al., 2020), but mostly in education (Hernández-Sellés et al., 2019). It supports the creation of both exploratory models and confirmatory analyses. PLS-SEM also works well for building complex models, forecasting, and evaluating the relationships between latent components. Small samples can be managed well, and normalization testing is not necessary (Hair et al., 2019). The PLS-SEM modeling multivariate method, which is utilized in our empirical investigation through the usage of the specialized program SmartPLS version 3.3.3 (Hair et al., 2019), is based on variance as the estimate method. A two-part assessment process is implied by the PLS-SEM methodology, with the first phase focusing on the measurement model and the second on the structural model (Hair et al., 2019). The model validation in the first phase is managed by taking into account the dependability and validity of the components and the manifest variables that are allocated to them (Hair et al., 2019). This approach entails calculating the hetero trait-mono trait ratio (HTMT), average variance extracted (AVE), composite reliability (CR), outer loadings, and Cronbach’s alpha () (Hair et al., 2019). In reflective models, the outer loadings are employed to examine the relationships between constructs and indicators. CA and CR are the metrics for inner consistency reliability (Hair et al., 2019). Since HTMT (Henseler et al., 2015) conducts a statistical discriminant validity check, AVE (Fornell and Larcker, 1981) quantifies the convergent efficiency of the factor degree. The values of all predictor constructs are shown by the inner VIF values, which point to a complementary test known as collinearity evaluation. The structural model validation, or second phase, determines the level of significance of the correlations between constructs by evaluating the presented hypotheses. The structural model’s path coefficients, value of *p*s, and *t*-values are calculated at this level. Multi-group analyses are used to validate each control variable, first at the global level and then among data subsets. The level of fit of the model is determined by the standardized root mean square residual (SRMR) measurement (Henseler et al., 2015). However, if there are no credible outputs for the assessment of the inner model’s predictive potential, then all indicators and actions taken up to this point from both stages are meaningless (Hair et al., 2019). The final endogenous variable’s R2 and F2 values are calculated for this purpose using the PLS predict algorithm.

### The measurement model assessment

The values of the measures, CR, AVE, and outer loading that characterize the convergent validity and inner consistency test for the reflective variables are shown in Tables 2, 3. We see that the outside loadings are higher than the 0.7-percent minimal limit (Hair et al., 2019). In turn, this validates the indication reliability. Every composite reliability value and the value are significantly higher than the reference value of 0.7 (Hair et al., 2019). This demonstrates the internal consistency of all constructs. All AVE values are higher than the threshold of 0.5 [164], confirming the model’s convergent validity.

**Table 2:** Validity and reliability, α, CR, and AVE.

	AUM	BIU	PEU	PEN	PU	SID	SIN	SPR	SSP	SS	α	CR	AVE
Actual use of M-learning	0.805										0.816	0.880	0.647
Behavioral intention to use M-learning	0.632	0.823									0.841	0.894	0.678
Perceived ease of use	0.579	0.586	0.804								0.819	0.880	0.647
Perceived enjoyment	0.634	0.656	0.650	0.917							0.905	0.940	0.840
Perceived usefulness	0.624	0.701	0.694	0.696	0.828						0.846	0.897	0.685
Social identity	0.371	0.277	0.477	0.491	0.414	0.872					0.843	0.905	0.760
Social interaction	0.502	0.555	0.546	0.621	0.568	0.319	0.901				0.884	0.928	0.812
Social presence	0.402	0.516	0.622	0.524	0.574	0.351	0.467	0.793			0.803	0.871	0.629
Social space	0.302	0.253	0.263	0.339	0.324	0.214	0.231	0.227	0.830		0.849	0.898	0.689
Students’ satisfaction	0.597	0.516	0.533	0.697	0.570	0.341	0.484	0.478	0.240	0.959	0.978	0.983	0.920

**Table 3:** Loading and cross-loading of measures.

	AUM	BIU	PEN	PEU	PU	SID	SIN	SPR	SS	SSP
										
AUM2	**0.878**	0.500	0.546	0.469	0.521	0.355	0.474	0.324	0.484	0.287
AUM3	**0.831**	0.548	0.567	0.495	0.558	0.282	0.454	0.342	0.587	0.303
AUM4	**0.786**	0.429	0.455	0.427	0.445	0.338	0.332	0.264	0.414	0.166
BIU1	0.495	**0.856**	0.545	0.547	0.617	0.272	0.522	0.471	0.411	0.258
BIU2	0.470	**0.801**	0.493	0.446	0.527	0.152	0.469	0.407	0.409	0.236
BIU3	0.577	**0.827**	0.588	0.488	0.557	0.292	0.425	0.381	0.458	0.174
BIU4	0.533	**0.808**	0.530	0.447	0.603	0.188	0.413	0.440	0.421	0.169
PEN1	0.605	0.616	**0.920**	0.597	0.656	0.464	0.581	0.461	0.627	0.322
PEN2	0.589	0.612	**0.935**	0.582	0.640	0.435	0.571	0.500	0.652	0.315
PEN3	0.548	0.576	**0.894**	0.609	0.618	0.450	0.554	0.480	0.638	0.295
PEU1	0.506	0.506	0.572	**0.823**	0.751	0.417	0.470	0.470	0.489	0.256
PEU2	0.489	0.465	0.546	**0.826**	0.577	0.363	0.450	0.398	0.388	0.275
PEU3	0.431	0.489	0.510	**0.798**	0.452	0.371	0.423	0.575	0.404	0.151
PEU4	0.427	0.418	0.451	**0.768**	0.406	0.379	0.408	0.576	0.429	0.148
PU1	0.524	0.597	0.652	0.563	**0.845**	0.283	0.586	0.491	0.516	0.305
PU2	0.537	0.627	0.567	0.482	**0.779**	0.349	0.463	0.388	0.428	0.266
PU3	0.471	0.555	0.497	0.557	**0.831**	0.354	0.401	0.516	0.441	0.233
PU4	0.530	0.540	0.579	0.690	**0.854**	0.390	0.422	0.505	0.496	0.265
SID1	0.336	0.252	0.452	0.416	0.369	**0.899**	0.302	0.301	0.305	0.177
SID2	0.275	0.224	0.392	0.370	0.330	**0.854**	0.277	0.289	0.263	0.174
SID3	0.351	0.246	0.436	0.454	0.381	**0.862**	0.257	0.326	0.318	0.207
SIN1	0.401	0.474	0.530	0.462	0.461	0.250	**0.886**	0.365	0.365	0.206
SIN2	0.487	0.553	0.590	0.512	0.533	0.309	**0.929**	0.406	0.439	0.230
SIN3	0.464	0.470	0.555	0.500	0.538	0.300	**0.888**	0.485	0.496	0.188
SPR1	0.317	0.434	0.422	0.581	0.465	0.297	0.376	**0.812**	0.358	0.152
SPR2	0.298	0.361	0.394	0.567	0.449	0.337	0.378	**0.855**	0.400	0.153
SPR3	0.292	0.403	0.370	0.398	0.440	0.185	0.338	**0.773**	0.363	0.190
SPR4	0.375	0.448	0.481	0.398	0.472	0.282	0.389	**0.727**	0.400	0.238
SS1	0.560	0.471	0.675	0.499	0.549	0.317	0.464	0.488	**0.958**	0.218
SS2	0.587	0.522	0.686	0.514	0.555	0.316	0.474	0.454	**0.977**	0.239
SS3	0.580	0.503	0.647	0.515	0.526	0.307	0.462	0.440	**0.955**	0.211
SS4	0.603	0.510	0.689	0.544	0.537	0.348	0.472	0.421	**0.956**	0.217
SS5	0.530	0.467	0.644	0.483	0.565	0.345	0.445	0.491	**0.949**	0.267
SSP1	0.260	0.235	0.301	0.239	0.256	0.153	0.215	0.206	0.203	**0.828**
SSP2	0.221	0.207	0.280	0.225	0.259	0.176	0.239	0.160	0.221	**0.858**
SSP3	0.214	0.176	0.231	0.168	0.281	0.225	0.159	0.160	0.113	**0.778**
SSP4	0.301	0.220	0.309	0.235	0.281	0.162	0.153	0.222	0.252	**0.853**

The interval [0.254, 0.830] encompasses all HTMT values that demonstrate discriminant validity, satisfying the conservative requirement that they must be less than 0.85 (Henseler et al., 2015). This is reflected in Table 4, which supports the claim that each construct is unique from the others in accordance with the criteria of empirical research (Hair et al., 2019; see Table 4).

**Table 4:** Discriminant validity evaluation for the reflective variables by HTMT criterion.

	AUM	BIU	PEU	PEN	PU	SID	SIN	SPR	SSP
Actual use of M-learning									
Behavioral intention to use M-learning	0.756								
Perceived ease of use	0.702	0.702							
Perceived enjoyment	0.732	0.751	0.751						
Perceived usefulness	0.745	0.830	0.815	0.792					
Social identity	0.446	0.325	0.569	0.56	0.490				
Social interaction	0.584	0.643	0.638	0.692	0.652	0.368			
Social presence	0.496	0.632	0.762	0.617	0.699	0.420	0.552		
Social space	0.355	0.300	0.308	0.385	0.382	0.254	0.267	0.279	
Students’ satisfaction	0.661	0.568	0.593	0.741	0.625	0.373	0.517	0.542	0.261

The VIF scores for all construct combinations are displayed in Table 5. The greatest value, which falls under the conservative upper limit of 3 (Becker et al., 2015), is 2.354. Therefore, no issues with predictor construct collinearity were found.

**Table 5:** Collinearity evaluation between the predictor constructs by inner VIF values.

	AUM	BIU	PEU	PEN	PU	SID	SIN	SPR	SSP	SS
Actual use of M-learning										
Behavioral intention to use M-learning	1.671									1.964
Perceived ease of use	1.712	1.928			2.298					
Perceived enjoyment			2.163		2.354					
Perceived usefulness		1.928								1.964
Social identity			1.343		1.403					
Social interaction			1.703		1.755					
Social presence			1.469		1.728					
Social space			1.137		1.138					
Students’ satisfaction	1.533									

### R^2^

According to Table 6’s (*R^2^*) results, the PEU, BIU, and SS account for 52% of the variance in actual M-learning use. Additionally, PU and BIU account for 35% of the variation in students’ satisfaction. According to Table 5, the PU and PEU account for 51% of the variation in the behavioral intention to use machine learning. Furthermore, SIN, SPR, SSP, SID, PEN, and PEU account for 61% of the variation in perceived usefulness. Furthermore, it is found that 56 percent of the variation in perceived ease of use for mobile learning is accounted for by SIN, SPR, SSP, SID, and PEN. The results of the study show that the R2 has a range of 0 to 1, with 0.25 being weak, 0.50 being moderate, and 0.75 being large (Hair et al., 2019). In Table 6, the R2 result is presented. The values obtained are satisfactory and have a significant or considerable impact on the actual use of mobile learning, behavioral intention to use mobile learning, perceived ease of use, perceived utility, and students’ satisfaction.

**Table 6:** Summary coefficient of determination, *R*^2^.

	*R* ^2^	*R*^2^-adjusted	Remark *R*^2^
Actual use of M-learning	0.525	0.522	Substantial
Behavioral intention to use M-learning	0.510	0.508	Substantial
Perceived ease of use	0.565	0.559	Substantial
Perceived usefulness	0.612	0.606	Substantial
Students’ satisfaction	0.351	0.348	Moderate

### The predictive relevance and effect size (F^2^)

The ^f2^ effect size is used to test the effect sizes of the outcome variables (Table 7). 0.35, 0.15, and 0.02 are acknowledged as having large, medium, and moderate effects, respectively (Hair et al., 2019). Cohen (2013) went on to say that values less than 0.02 have no impact. Table 7 displays the effect size of pathways ranging from no effect to a considerable influence based on these characteristics.

**Table 7:** The result of (*F*^2^).

	AUM	BIU	PEU	PEN	PU	SID	SIN	SPR	SSP	SS
Actual use of m-learning										
Behavioral intention to use M-learning	0.156									0.242
Perceived ease of use	0.255	0.239			0.211					
Perceived enjoyment			0.293		0.111					
Perceived usefulness		0.340								0.131
Social identity			0.145		0.213					
Social interaction			0.231		0.019					
Social presence			0.176		0.123					
Social space			0.201		0.128					
Students’ satisfaction	0.126									

### The structural model assessment

**Figure 2:**
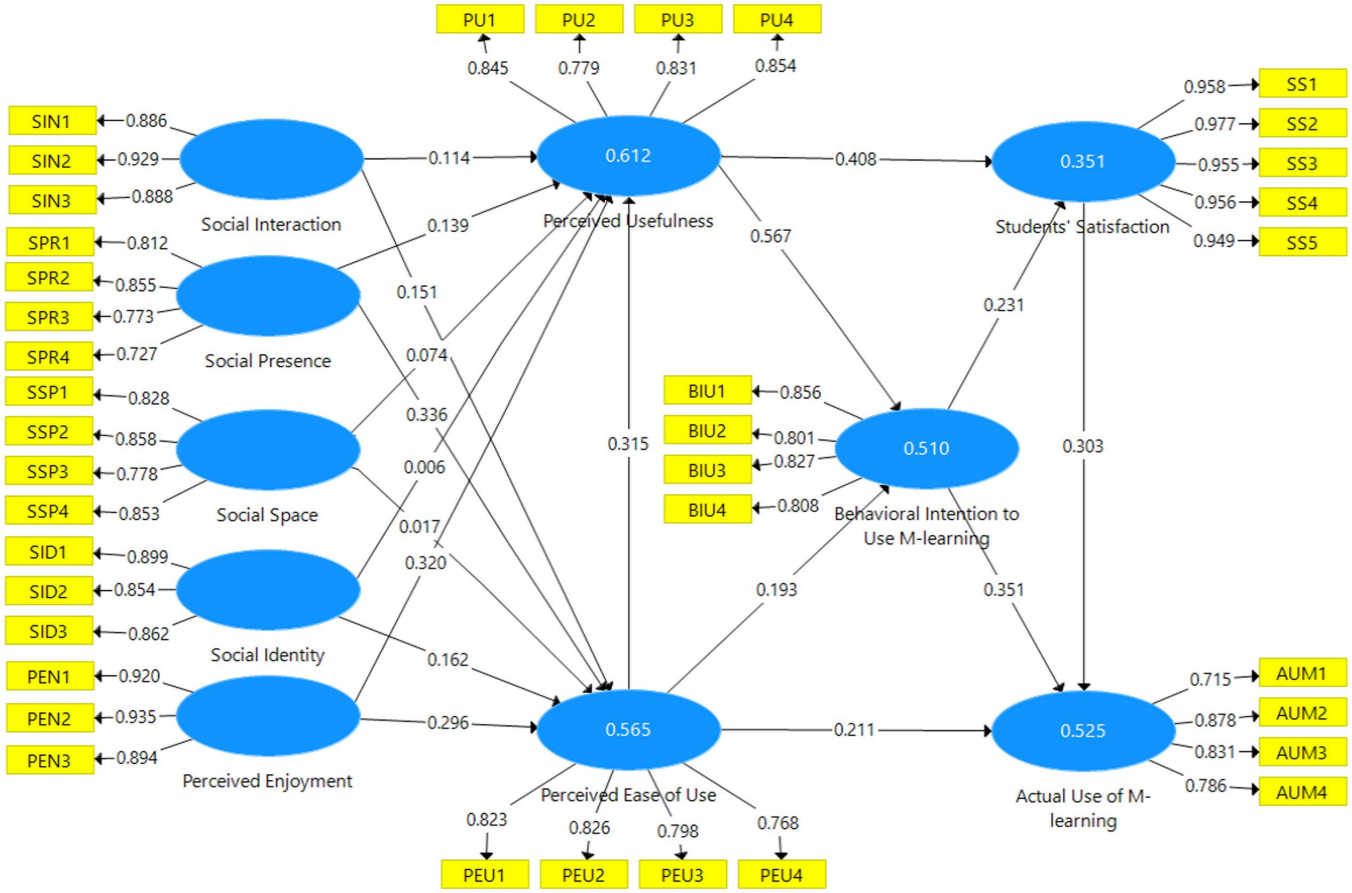
The findings for path coefficient.

By evaluating the structural model and model fit using a variety of metrics, hypotheses were tested. The procedure would specify the route coefficient, *t*-value, value of p, and mediating effects in order to decide whether or not the hypothesis was accepted. The development of the structural model assessment in this study to link endogenous variables (such as perceived usefulness, perceived ease of use, behavioral intention to use mobile learning, SS, and actual use of mobile learning) to exogenous variables is shown in Figure 2 (such as social interaction, social presence, social space, social identity, and perceived enjoyment).

Table 8 with Figure 2 and shows that the t-value amongst the factors influencing Social Interaction (β = 0.114, *t* = 2.275, *p* < 0.05), Social Presence (β = 0.139, *t* = 3.245, *p* < 0.05), Social Space (β = 0.074, *t* = 2.375, *p* < 0.05), Perceived Enjoyment (β = 0.320, *t* = 6.321, *p* < 0.05) showed significant effects to Perceived Usefulness. Thus, H1, H3, H5, and H9 are accepted. However, Social Identity (β = 0.006, *t* = 0.124, *p* < 0.05) for H7 were rejected because there were no significant effects towards Perceived Usefulness. The SEM results revealed a direct significant relationship between Social Interaction (β = 0.151, *t* = 3.323, *p* < 0.05), Social Presence (β = 0.336, *t* = 5.431, *p* < 0.05), Social Identity (β = 0.162, *t* = 3.908, *p* < 0.05), Perceived Enjoyment (β = 0.296, *t* = 4.687, *p* < 0.05) and Perceived Ease of Use. Thus, H2, H 4, H8, and H10 were supported by the model. Additionally, the SEM results revealed no direct significant relationships between Social Space (β = 0.017, *t* = 0.527, *p* < 0.05) and Perceived ease of use. Thus, the hypothesis six H6 was rejected by the model. The results also indicated that perceived ease of use (PEU) has a significantly positive effect on perceived usefulness (PU) (β = 0.315, *t* = 6.026, *p* < 0.05), behavioral intention to use M-learning (BIU) (β = 0.193, *t* = 3.199, *p* < 0.05), and actual use of M-learning (AUM) (β = 0.211, *t* = 4.364, *p* < 0.05) with these results supporting hypotheses H11, H12 and H13. We also found that Perceived Usefulness (PU) has a significantly positive effect on Students’ Satisfaction (SS) (β = 0.408, *t* = 6.095, *p* < 0.05), and behavioral intention to use M-learning (BIU) (β = 0.567, *t* = 10.078, *p* < 0.05), with this result supporting H14 and H15. In addition, behavioral intention to use M-learning (BIU) has a significant effect on both Students’ Satisfaction (SS) (β = 0.231, *t* = 3.473, *p* < 0.05) and actual use of M-learning (AUM) (β = 0.351, *t* = 7.311, *p* < 0.05) respectively, with this result supporting H16and H17. Finally, students’ satisfaction (SS) (β = 0.303, *t* = 6.986, *p* < 0.05) showed significant effects actual use of M-learning Thus, H18 is accepted.

**Table 8:** Summary of hypotheses testing results.

Factors	H	*β*	*T*-values	*P*-values
Social interaction → Perceived usefulness	H 1	0.114	2.275	0.023
Social interaction → Perceived ease of use	H 2	0.151	3.323	0.001
Social presence → Perceived usefulness	H 3	0.139	3.245	0.001
Social presence → Perceived ease of use	H 4	0.336	5.431	0.000
Social space → Perceived usefulness	H 5	0.074	2.375	0.018
Social space → Perceived ease of use	H 6	0.017	0.527	0.598
Social identity → Perceived usefulness	H 7	0.006	0.124	0.902
Social identity → Perceived ease of use	H 8	0.162	3.908	0.000
Perceived enjoyment → Perceived usefulness	H 9	0.320	6.321	0.000
Perceived enjoyment → Perceived ease of use	H 10	0.296	4.687	0.000
Perceived ease of use → Perceived usefulness	H 11	0.315	6.026	0.000
Perceived ease of use → Behavioral intention to use M-learning	H 12	0.193	3.199	0.001
Perceived ease of use → Actual use of M-learning	H 13	0.211	4.364	0.000
Perceived usefulness → Students’ satisfaction	H 14	0.408	6.095	0.000
Perceived usefulness → Behavioral intention to use M-learning	H 15	0.567	10.078	0.000
Behavioral intention to use M-learning → Students’ satisfaction	H 16	0.231	3.473	0.001
Behavioral intention to use M-learning → Actual use of M-learning	H 17	0.351	7.311	0.000
Students’ satisfaction → Actual use of M-LEARNING	H 18	0.303	6.986	0.000

## Discussion and consequences

The purpose of this study is to identify the variables that influence King Saud University students’ adoption of mobile learning. The end product is a social cognition theory and TAM model-based theoretical framework for m-learning. The suggested study structure was put to the test using a randomly chosen sample of King Saud University students. The findings are positively and significantly related to each predictor, as well as to how well students are doing with M-learning and how much they actually utilize it. The findings of the regression analysis and the evaluation of the structural model are both substantial and have an impact on every component taken into account.

According to the research, the adoption of mobile learning systems is influenced by a number of variables, including user satisfaction, organizational considerations, quality features, and technological concerns. We therefore wanted to look at the key variables that can influence how mobile learning solutions are actually used. This study proposed a new model in order to accomplish this goal by including new variables such as social interaction, social presence, social space, social identity, perceived enjoyment, perceived ease of use, perceived usefulness, behavioral intention to use mobile learning, SS, and actual use of mobile learning. The TAM model and social cognitive theory were also used in this study to describe the key elements that govern how mobile learning systems are actually used. To assess the hypotheses, structural equation modeling (SEM) was performed. The 18 hypotheses were supported by the study’s findings. The findings also showed that the proposed research model can account for 52.5% of the variation in how mobile learning systems are actually used. Following is a discussion of the study’s results.

The findings are consistent with those of (Turner et al., 1987; Jung et al., 2002; Kreijns et al., 2004; Van der Heijden, 2004; Miyazoe and Anderson, 2010; Joksimović et al., 2015) and show that social contact, social presence, social space, social identity, and perceived enjoyment significantly influence perceived utility and ease of use. The model analysis also demonstrates that perceived usefulness and ease of use have a positive effect on SS and actual use of mobile learning. His results differ from those of (Behera and Purulia, 2013; Badwelan et al., 2016), but they are compatible with those of (Davis et al., 1989; Venkatesh, 2000; Al-Rahmi et al., 2017; Kumar Basak et al., 2018). The results also show that perceived utility and ease of use have a significant impact on the positive behavioral intention to use M-learning. This finding is consistent with those of (Wang et al., 2009; Al-Rahmi et al., 2018b; Alowayr and Al-Azawei, 2021; Ullah et al., 2021).

Therefore, pupils are willing to accept M-learning for educational purposes since they feel satisfied with using mobile learning. Numerous researchers have examined the significance of PU and PEU in the context of M-learning (Kumar Basak et al., 2018). This study’s findings corroborate those of previous researchers (Aremu and Adeoluwa, 2021; Hoi and Mu, 2021). Two crucial components of the TAM educational paradigm are also seen in the findings of other researchers, such as (Alghazi et al., 2020). Students use M-learning to improve their education (Davis et al., 1989). This might be the case because students are content with using the computerized M-learning version and have favorable opinions of the system’s operation.

Based on the findings in Figures 1, 2, two of the 18 hypotheses were rejected, while 16 of them were accepted in this study model, which consisted of 10 components that all had a significant and positive impact on the quality of mobile learning applications. These findings demonstrate the value of utilizing mobile learning tools that meet students’ requirements for usability and usability in fostering social engagement. The outcomes also demonstrate that the achievement of social presence is positively impacted by using mobile learning applications that meet students’ criteria for usability and ease of use. And the results show that while social space is negatively impacted by simplicity of use, social space is favorably impacted by employing mobile learning applications that satisfy students’ needs for usefulness. The following data show that while social space is negatively impacted by usefulness, the accomplishment of social identity is positively impacted by employing mobile learning applications that satisfy students’ needs for ease of use. This is different from earlier findings (Behera and Purulia, 2013; Badwelan et al., 2016). The findings demonstrate that using mobile learning tools that meet students’ criteria for usability and simplicity of use has a positive effect on achieving perceived satisfaction. According to the study’s findings, using mobile learning tools that meet students’ needs for utility, behavioral intentions to use m-learning, and actual m-learning use has a positive effect on perceived ease of use. The findings of this study also demonstrate the positive effects of using mobile learning applications that meet students’ requirements and behavioral intentions to use m-learning on perceived usefulness achievement. Additionally, the results of this study show that employing mobile learning applications that satisfy students and encourage them to use m-learning significantly impacts the achievement of behavioral intention to do so. Finally, the results of this study show that employing mobile learning applications that correspond to students’ actual usage of m-learning has a beneficial impact on the attainment of students’ satisfaction.

These findings imply that users’ happiness and subsequent use of mobile learning systems will rise when they believe the materials and contents of these systems are adequate, thorough, and support a variety of learning activities such PowerPoint slides, assignments, and tests. According to this study, functionality has a considerable and advantageous impact on students’ satisfaction with mobile learning solutions. This illustrates that when a mobile learning system provides the characteristics necessary for instructional activities, student happiness will increase. These results are consistent with e-learning study by (Badwelan et al., 2016; Al-Emran et al., 2018; Kumar Basak et al., 2018), which discovered that the functionality of an m-learning system had a positive impact on students’ satisfaction.

### Research contributions

Both theoretical and practical advancements are made through this investigation. By offering a novel model that captures the most important factors influencing M-learning adoption among students in public Saudi universities, this study adds to the body of knowledge on the topic from a theoretical standpoint. Second, this study makes it clear that crucial elements like social interaction, social presence, social space, social identity, perceived enjoyment, perceived ease of use, and perceived usefulness were crucial in raising behavioral intention to use mobile learning, SS, and actual use of mobile learning. These elements will also ensure that the learning process is sustained through the use of this distance learning tool. Third, this study demonstrates that it is appropriate to analyze the variables affecting students’ acceptance of M-learning using the integrated social cognitive theory and TAM model.

Regarding the study’s practical implications, the results can aid Saudi institutions in better comprehending the procedure for implementing M-learning projects. To encourage student adoption of M-learning systems, universities should take into account critical elements of social interaction, social presence, social space, and social identity in addition to perceived fun, perceived ease of use, and perceived value. The results of this study will help university decision-makers, designers, and developers make sure that students actively use M-learning platforms. As a result, the statement that follows best sums up the importance of this study: This study explains the importance of educational environmental aspects in improving the quality of mobile learning systems, which could improve learning efficacy and student performance and were not included in other studies on mobile learning. It does this by integrating social cognition theory with the TAM model.

### Limitations of research

No matter how much this research contributes, its flaws must be fixed. The work’s limitations have had an impact on the study’s findings as well. In order to increase the sample size and see whether students from other colleges can demonstrate equivalent results, more study is first needed. Second, in addition to the aspects discussed in the study, future research should consider a variety of other factors that may influence the willingness to use mobile learning, such as engagement tools, quality of design, system quality, quality of service, and social impact. Institutional benefits and other factors including company culture, strategy, and leadership may also have an impact on the effectiveness of mobile learning. Future research could look into their outcomes.

### Conclusions and future work

In this study, an unique model based on the integrated social cognition theory and the TAM model was constructed in order to measure students’ satisfaction with the actual use of M-learning systems in higher education. The suggested model was empirically assessed using the SEM method. The results of this investigation supported 18 of the original hypotheses; 16 of them were accepted, while 2 were rejected. The findings also showed that the proposed study model could account for 52.5% of the variation in actual mobile learning system usage. The relationship between behavioral intention to use mobile learning, SS, and actual use of mobile learning systems is made clear by this finding, which also highlights the influence of social interaction, social presence, social space, social identity, perceived enjoyment, perceived ease of use, and perceived usefulness factors. A thorough review of the literature served as the foundation for the creation of the new paradigm for M-learning throughout the world, including in Saudi Arabia’s higher education system. The inquiry into students’ satisfaction and practical use of M-learning systems in higher education is mostly based on the 10 constructs generated from the social cognitive theory and the TAM model. This research strongly implies that universities employ the TAM model and social cognition theory to persuade students to adopt M-learning for educational goals. They are the first to believe that they make a significant impact. Additionally, the study shows that the conclusions are based on King Saud University student viewpoints, which may or may not be indicative of the current status of the world. Future studies should explore the TAM model and social cognition theory’s planning guidelines in light of the expanding usage of M-learning, as well as assess their potential for use in educational settings. Future studies in this area should explore how M-learning is viewed by educators and other stakeholders in higher education. Finally, extending the study’s findings and comparing viewpoints with those of other countries might help us better understand how prospects for M-learning in higher education can be handled.

## Data availability statement

The original contributions presented in the study are included in the article/Supplementary material, further inquiries can be directed to the corresponding author.

## Ethics statement

Ethical review and approval was not required for the study on human participants in accordance with the local legislation and institutional requirements. Written informed consent from the patients/ participants or patients/participants legal guardian/next of kin was not required to participate in this study in accordance with the national legislation and the institutional requirements.

## Author contributions

AA and NA: conceptualization, methodology, software, validation, formal analysis, investigation, resources, data curation, writing—original draft preparation, writing—review and editing, visualization, supervision, project administration, and funding acquisition. All authors contributed to the article and approved the submitted version. All authors contributed to the article and approved the submitted version.

## Funding

This work was supported by the King Saud University, Riyadh, Saudi Arabia, through Researchers Supporting Project RSP2022/R417.

## Conflict of interest

The authors declare that the research was conducted in the absence of any commercial or financial relationships that could be construed as a potential conflict of interest.

## Publisher’s note

All claims expressed in this article are solely those of the authors and do not necessarily represent those of their affiliated organizations, or those of the publisher, the editors and the reviewers. Any product that may be evaluated in this article, or claim that may be made by its manufacturer, is not guaranteed or endorsed by thepublisher.
