# Supplementary material for: The integrated social cognitive theory with the TAM model: The impact of M-learning in King Saud University art education
Source: Front Psychol. 2022 Nov 24;13:1050532. doi: 10.3389/fpsyg.2022.1050532 (PMC9731295; doi:10.3389/fpsyg.2022.1050532)
Supplement: Supplementary file 1 [file Table_1.DOCX]

**Appendix**

| ***Social Interaction*** | |
| --- | --- |
| 1 | I often discussed learning issues with others in the art education course via mobile learning. |
| 2 | I often shared learning materials or opinions with others in the art education course via mobile learning. |
| 3 | I often talked about learning goals and tasks with others in the art education course via mobile learning. |
| ***Social Presence*** | |
| 1 | I felt comfortable participating in the art education course discussions via mobile learning. |
| 2 | I felt comfortable interacting with other participants in the art education course via mobile learning. |
| 3 | I felt that my point of view was acknowledged by other participants in the art education course via mobile learning. |
| 4 | Discussions conducted via mobile learning tend to be more impersonal than face-to-face discussions. |
| ***Social Space*** | |
| 1 | I maintained contact with all other group members in the art education course via mobile learning. |
| 2 | Group members took the initiative to get in touch with others in the art education course via mobile learning. |
| 3 | Group members spontaneously started conversations with others in the art education course via mobile learning. |
| 4 | Group members asked others how the class was going in the art education course via mobile learning. |
| ***Social Identity*** | |
| 1 | I am a valuable member in art education courses because of my use of mobile learning. |
| 2 | Through mobile learning use, I believe that I am one of the art education course members. |
| 3 | Through mobile learning use, I am an important member of art education courses. |
| ***Perceived Enjoyment*** | |
| 1 | I find the mobile learning process enjoyable for learning an art education course. |
| 2 | The actual process of using mobile learning is pleasant for learning an art education course. |
| 3 | I am having fun using mobile learning to learn an art education course. |
| ***Perceived Usefulness*** | |
| 1 | Using mobile learning would enhance my effectiveness in studying. |
| 2 | Using mobile learning would improve my course performance. |
| 3 | Using mobile learning would improve my productivity in courses. |
| 4 | I find mobile learning useful for my studies. |
| ***Perceived Ease of Use*** | |
| 1 | I find mobile learning easy to use. |
| 2 | It would be easy for me to find the required information using mobile learning. |
| 3 | Mastering mobile learning would be easy for me. |
| 4 | My interaction with mobile learning is clear and understandable. |
| ***Behavioural Intention to Use mobile learning*** | |
| 1 | I intend to use mobile learning during an art education course. |
| 2 | I will return to mobile learning often to learn an art education course. |
| 3 | I will continue to use mobile learning for learning an art education course. |
| 4 | I intend to use mobile learning frequently for learning an art education course. |
| ***Students satisfaction*** | |
| 1 | I was able to learn an art education course through mobile learning. |
| 2 | I was able to learn about art education courses from discussions through mobile learning. |
| 3 | I was stimulated to do additional reading or research on art education courses through mobile learning. |
| 4 | I am effective at knowledge exchange because I use mobile learning. |
| 5 | Overall, I am satisfied with learning art education courses through mobile learning. |
| ***Actual Use mobile learning*** | |
| 1 | I use mobile learning frequently for learning an art education course. |
| 2 | I tend to use mobile learning frequently for learning an art education course. |
| 3 | I spend a lot of time researching mobile learning for an art education course. |
| 4 | I get involved a lot with mobile learning for learning an art education course. |
